# Predictive Factors of Concerns about Falling in People with Parkinson's Disease: A 3-Year Longitudinal Study

**DOI:** 10.1155/2019/4747320

**Published:** 2019-12-12

**Authors:** Magnus Lindh-Rengifo, Stina B. Jonasson, Niklas Mattsson, Susann Ullén, Maria H. Nilsson

**Affiliations:** ^1^Department of Health Sciences, Lund University, Lund, Sweden; ^2^Memory Clinic, Skåne University Hospital, Malmö, Sweden; ^3^Clinical Memory Research Unit, Department of Clinical Sciences Malmö, Lund University, Lund, Sweden; ^4^Department of Neurology, Skåne University Hospital, Lund, Sweden; ^5^Wallenberg Centre for Molecular Medicine, Lund University, Lund, Sweden; ^6^Clinical Studies Sweden—Forum South, Skåne University Hospital, Lund, Sweden

## Abstract

**Introduction:**

Fear of falling (FOF) is more common in people with Parkinson's disease (PD) than in healthy controls. It can lead to several negative consequences such as restrictions in everyday life. Moreover, FOF is a risk factor for future falls.

**Aim:**

This study aimed to identify predictive factors of FOF (conceptualized as concerns about falling) after three years, with and without adjusting for concerns about falling at baseline, in people with PD.

**Methods:**

This study included 151 participants (35% women) with PD. At baseline, their mean (SD) age and PD duration were 68 (±9.0) and 9 (±6.1) years, respectively. The Falls Efficacy Scale-International (FES-I) was used as the dependent variable in multivariable linear regression analyses.

**Results:**

The mean (SD) FES-I score increased from 28.1 (11.9) to 33.1 (14.0) three years later (*p* < 0.001). The strongest (according to the standardized regression coefficient, *β*) predictor of concerns about falling was walking difficulties (*β* = 0.378), followed by age (0.227), problems maintaining balance while dual tasking (0.172), and needing help in daily activities (0.171). When adjusting for baseline FES-I scores, the strongest predictive factor was problems maintaining balance while dual tasking (*β* = 0.161), which was followed by age (0.131) and female sex (0.105).

**Conclusions:**

This study pinpoints several predictive factors of concerns about falling that are modifiable and which could be addressed in rehabilitation: perceived walking difficulties, having problems maintaining balance while dual tasking, and dependence on others in daily activities. The importance of dual tasking is a novel finding, which future studies need to confirm or refute. One should be aware of the fact that an increased age predicts concerns about falling with and without adjusting for baseline FES-I scores, whereas female sex predicts concerns about falling only when adjusting for baseline FES-I scores.

## 1. Introduction

Fear of falling (FOF) is considered a broad term comprised of different conceptualizations, such as concerns about falling [1, 2], low fall-related self-efficacy [3], a loss of balance confidence [4], and fall-related activity avoidance [5]. FOF is more common in people with Parkinson's disease (PD) than in healthy controls [6, 7]. The prevalence ranges between 37–55% in people with PD [8–11]. It is more prevalent among those with a history of falls, but it is also reported by people without prior falls [12]. Several PD studies have described negative consequences of FOF, which has been shown to be a risk factor for future falls [13] and a barrier for engaging in exercise [14]. FOF can restrict everyday life [15] and result in activity limitations [16]. Moreover, it is negatively associated with perceived participation [17] and quality of life [18].

In order to be able to design efficient interventions for FOF in people with PD, it is important to gain an increased understanding of predictive factors of FOF. Seven previous cross-sectional studies used multivariable analyses in order to identify factors associated with FOF in people with PD [8, 10, 19–23]. Four out of the seven studies addressed fall-related self-efficacy [8, 10, 20, 21]. Several factors were shown to be significantly associated with fall-related self-efficacy: PD severity [21]; PD duration [21]; severity of parkinsonian motor symptoms [21]; perceived walking difficulties [8, 10]; functional balance performance [10]; turning hesitations [8]; dependence for daily activities [8, 10, 20]; depressive symptoms [21]; cognitive functioning [21]; and fatigue [8, 10] and motor fluctuations [8]. Another study concerned balance confidence instead of fall-related self-efficacy and identified postural instability, gait difficulties, and knee muscle strength as associated factors [19]. Two of the cross-sectional studies addressed concerns about falling [22, 23]. Jonasson et al. [22] used the same cohort as in the present study and showed that perceived walking difficulties, orthostatism, motor symptoms, age, and fatigue were significantly associated with concerns about falling. Franzén et al. [23] identified depressive symptoms, balance performance, and the use of mobility devices as associated factors. These two studies [22, 23] showed conflicting results in relation to the use of mobility devices, depressive symptoms, motor symptoms, and age.

Longitudinal studies are necessary to clarify the most important factors leading to FOF. To our knowledge, there is only one longitudinal study that addressed predictors of FOF in people with PD [24]. That study (2-year follow-up, *N* = 88) addressed a change in fall-related self-efficacy and presented two different models; the first showed that the total number of falls was the only significant predictor, whereas the second found PD severity to be the only significant predictor. There is a need for larger studies with a longer follow-up to establish predictive factors of FOF in PD; such knowledge would be valuable for identifying factors of importance for future intervention studies.

This study aimed to identify predictive factors of FOF (conceptualized as concerns about falling) after three years, with and without adjusting for concerns about falling at baseline, in people with PD.

## 2. Materials and Methods

This study is part of a longitudinal project “Home and Health in People Ageing with PD” (PI Nilsson, MH). Baseline assessments were conducted in 2013 and a 3-year follow-up in 2016. More information about the methods and design of the larger project is described in the study protocol [25].

### 2.1. Participants and Recruitment

**Figure 1:**
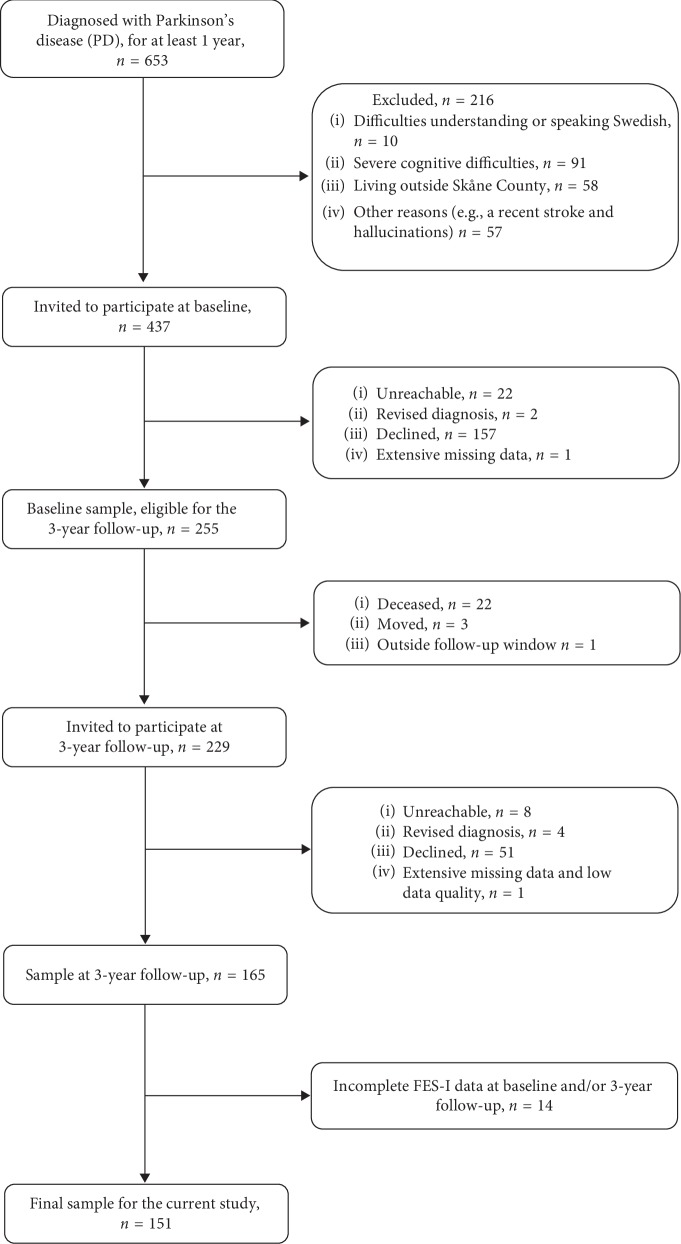
Flowchart: participant recruitment process.

Outpatient participants were recruited from three hospitals in southern Sweden (see Figure 1, which presents a flowchart of the recruitment process). Details of recruitment and follow-up procedures have been described before, i.e., for baseline [26] and the 3-year follow-up [27]. At baseline, 653 persons met the inclusion criterion of a PD diagnosis (ICD10-code G20.9), since at least one year. Of those, 216 were not eligible due to difficulties in understanding or speaking Swedish (*n* = 10), severe cognitive difficulties (*n* = 91), living outside Skåne County (*n* = 58), or other reasons that made them unable to give informed consent or take part in the majority of the data collection (e.g., hallucinations, a recent stroke; *n* = 57). The remaining 437 persons were invited to participate. Of these, 22 were unreachable, two had a revised diagnosis, and 157 declined. One person was excluded due to extensive missing data, resulting in a sample of 255 participants at baseline.

All those who completed baseline assessments and had agreed to be contacted again (*n* = 255) were considered eligible for the 3-year follow-up (±3 months). At that time, 22 persons were deceased, three had moved, and one ended up outside the follow-up window. Thus, 229 persons were invited to participate. Of these, eight were unreachable, four had a revised diagnosis, and 51 declined. One person was excluded due to extensive missing data and low data quality. In total, the 3-year follow-up had a sample of 165 persons. Since the primary focus of this study was FOF, i.e., conceptualized as concerns about falling and assessed by using the Falls Efficacy Scale-International (FES-I), we only included subjects with a total score of FES-I at both time points (*N* = 151 of the total 165). At baseline, their mean (SD) age was 68 (±9.0) years, and 35.1% were female. Mean PD duration at baseline was 9 (±6.1) years. Median (*q*1–*q*3) PD severity during “on-state” was 2 (2-3; assessed by using the Hoehn & Yahr staging, rated 1–5, higher = worse) [28]. Additional descriptive information is presented in Table 1.

The included 151 participants were significantly younger and had a shorter PD duration than the 104 who were lost for follow-up: mean 68 vs. 72 years old at baseline, *p* < 0.001 and mean 9 vs. 10 years PD duration, *p*=0.048 (independent samples *t*-tests). Although a trend, there was no statistically significant difference regarding sex (Pearson's Chi-square test: 35% vs. 47% woman, *p*=0.068).

### 2.2. Ethics

The project was conducted in accordance with the Helsinki Declaration and was approved by the Regional Ethical Review Board in Lund, Sweden (nos. 2012/558 and 2015/611). All participants gave their written informed consent.

### 2.3. General Procedure

The data collectors underwent project-specific training. Two registered occupational therapists collected the data during baseline. Two other occupational therapists, with the help of a PhD student in physiotherapy, collected data during the 3-year follow-up. Both data collection waves comprised a self-administered postal survey and a home visit, which included structured interviews and clinical assessments. The postal survey was administered about ten days ahead of the home visit.

### 2.4. Data Collections

Concerns about falling constituted the dependent variable and was assessed by using the self-administered questionnaire Falls Efficacy Scale-International (FES-I), which includes 16 items (i.e., activities). The response options are not at all concerned (score 1), somewhat concerned (score 2), fairly concerned (score 3), or very concerned (score 4) [2]. The total score can range from 16–64 (higher = more concerns about falling). For descriptive purposes, we also included a dichotomous FOF (Yes/No) question: are you afraid of falling?

Several independent variables were considered. Data on personal factors included age, sex, and general self-efficacy (assessed with the self-administered General Self-Efficacy Scale, scored 10–40 points, higher = better) [29]. Environmental factors included living situation (alone/not alone) and the use of mobility devices outdoors. Several interview-administered questions addressed the use of different mobility devices, and responses were categorised into users and nonusers of any device.

Clinical assessments administered at the home visit addressed the severity of parkinsonian motor symptoms and cognitive functioning. The motor part of the Unified Parkinson's Disease Rating Scale (UPDRS III) is scored 0–108 points (higher = worse) [30]. The Montreal Cognitive Assessment (MoCA) is scored 0–30 points (higher = better) [31] (permission was granted for the use of the MoCA instrument).

Several self-administered questionnaires were included in the postal survey. Walking difficulties in daily life were assessed by using the Generic Walk-12 (Walk-12G, scored 0–42, higher = worse) [32]. Fatigue was assessed with the energy subscale of the Nottingham Health Profile (NHP-EN) [33]. Those who affirmed at least one out of its three dichotomous questions were classified as having fatigue [34]. Needing help from others in activities of daily living was assessed by using the Parkinson's disease Activities of Daily Living Scale (PADLS, scored 1–5) [35]; those who scored >2 were classified as needing help from others [9]. Freezing of gait was assessed by using item 3 of the self-administered version of the Freezing of Gait Questionnaire (FOGQsa) [36], which is scored 0–4 (higher = worse). Those who scored >0 were categoried as freezers [8]. Orthostatism was assessed by using the dichotomous item 20 of the Nonmotor symptoms questionnaire (NMSQuest) [37]. The self-administered survey also included a dichotomous (Yes/No) question that addressed the presence of motor fluctuations: “do you feel that the medical effect fluctuates during the day, with periodically increasing parkinsonian symptoms, e.g., when it is time for a new medical dose?” Another dichotomous (Yes/No) question concerned perceived balance problems while dual tasking: “do you experience balance problems while standing or walking when doing more than one thing at a time, e.g., carrying a tray while walking?”

Some rating scales and questions were administered as an interview at the home visit. The Geriatric Depression Scale (GDS-15) includes 15 items and is scored from 0 to 15 points (higher scores = more depressive symptoms) [38]. Dichotomous (Yes/No) questions addressed pain (are you troubled by pain?) and fall history during the past 6 months.

### 2.5. Statistical Analysis

The relationships among the independent variables were studied by using Pearson's correlation coefficient (*r*). There were no signs of multicollinearity (i.e., *r* > 0.7), except between Walk-12G and FES-I baseline scores (*r* = 0.865; FES-I baseline was included as a controlling variable in one of the regression models). Associations between the dependent variable (i.e., FES-I at the 3-year follow-up) and independent variables were analysed in a series of univariable linear regression analyses (Table 1). In order to avoid leaving out a confounding variable, we decided to include all variables with a *p* value below 0.3 into the following multivariable linear regression analyses (method: enter). All 17 independent variables fulfilled this criterion and were consequently included. The estimates and *p* value for each independent variable were examined, and the variable with the highest *p* value was manually removed from the multivariable model. This step continued until all remaining independent variables had *p* values <0.1. Finally, the analyses were re-ran and controlled for baseline FES-I scores in order to identify predictive factors of a change in FES-I scores over a 3-year period (i.e., Model 2). That is, this model identifies predictive factors given the FES-I score at baseline. Unadjusted and adjusted *R*^2^ are presented as indications of the predictive capacity of the models. Statistical significance was set to a 0.05 level.

All statistical analyses were performed using SPSS statistics, version 25 (IBM Corporation, Armonk, NY, United States).

## 3. Results

At baseline, 40% (61/151) of the participants reported FOF, which increased to 55% (83/151, *p* < 0.001) at the 3-year follow-up.

Total and item scores of FES-I are presented in Table 2. The mean (SD) FES-I score increased (*p* < 0.001) from 28.1 (11.9) to 33.1 (14.0) three years later. This increase exceeded the measurement error (i.e., SEM), see Table 2. At baseline, the top three activities that were rated as the most concerning were “walking on a slippery surface” (item 11, mean score 2.36), “walking on an uneven surface” (item 14, mean score 2.05), and “walking up or down a slope” (item 15, mean score 1.96). The same activities remained the top three at the 3-year follow-up.

### 3.1. Univariable Regression Analyses

Univariable linear regression analyses are presented in Table 1; FES-I scores at the 3-year follow-up was the dependent variable. Fifteen out of the 17 independent variables had a *p* value <0.05. Walking difficulties (Walk-12G) had the strongest effect on follow-up FES-I scores (*β* = 0.683, *p* < 0.001), followed by balance problems while dual tasking (*β* = 0.508, *p* < 0.001).

### 3.2. Multivariable Regression Analyses

#### 3.2.1. Model 1 (Unadjusted for Baseline FES-I Scores)

The final model resulted in six independent variables explaining 57.5% (adjusted *R*^2^) of the variance in FES-I scores at the 3-year follow-up. Four out of the six factors were statistically significant. The strongest predictive factor (as assessed by the standardized regression coefficients, *β*) was walking difficulties (*β* = 0.378) followed by age (*β* = 0.227), problems maintaining balance while dual tasking (*β* = 0.172), and needing help in daily activities (*β* = 0.171). The other two variables were depressive symptoms (*β* = 0.118) and sex (*β* = 0.107); both had a *p* value of 0.060. See Table 3 for further details.

#### 3.2.2. Model 2 (Adjusted for Baseline FES-I Scores)

When adjusting for FES-I scores at baseline, the final model included four independent variables explaining 62.6% (adjusted *R*^2^) of the variance in FES-I scores at the 3-year follow-up. The strongest predictive factor for a change in FES-I scores (when adjusting for baseline FES-I scores) was problems maintaining balance while dual tasking (*β* = 0.161), which was followed by age (*β* = 0.131) and female sex (*β* = 0.105). The fourth variable (i.e., needing help in daily activities) was not significantly associated with concerns about falling (*β* = 0.116, *p*=0.068). See Table 4 for further details.

## 4. Discussion

The main findings in this prospective longitudinal study were that (1) the strongest predictive factor for concerns about falling was perceived walking difficulties followed by age, problems maintaining balance while dual tasking, and needing help in daily activities and (2) the strongest predictor for a change in concerns about falling over a 3-year period (when adjusting for baseline FES-I scores) was problems maintaining balance while dual tasking, higher age, and (female) sex.

The fact that walking difficulties was the strongest predictor for concerns about falling is in line with prior cross-sectional studies [8, 10, 22]. This study adds to the body of knowledge due to its longitudinal design. That is, the present finding implies that perceived walking difficulties in daily life seem to be of importance for developing concerns about falling. However, perceived walking difficulties did not predict a change in concerns about falling. A plausible explanation for this is that baseline FES-I scores were then forced to remain in the multivariable model since we wished to address a change. Due to multicollinearity, Walk-12G could not contribute with any further predictive capacity when baseline FES-I scores were controlled for. Considering this relationship between concerns about falling and walking difficulties, walking difficulties might still play a role even though the analysis says otherwise. Several interventions have shown beneficiary effects on gait parameters in people with PD such as treadmill training [39] and different types of external cueing, e.g., auditory and visual cues [40]. One study showed that treadmill training had positive effects on maximum gait speed, total walking distance, and FOF (conceptualized as fall-related self-efficacy) [41]. The present study underscores the potential value of focusing on perceived walking difficulties to prevent the development of FOF in people with PD. As yet, few intervention studies have used perceived walking difficulties as their primary outcome.

Problems maintaining balance while dual tasking predicted concerns about falling and was the strongest predictor for a change in concerns about falling. This finding is in contrast to two cross-sectional studies [10, 22], whereof one included the same cohort as ours. The discrepancy might reflect different study designs, i.e., cross-sectional versus a follow-up study design. As yet, there is only one prior longitudinal study of predictive factors of a change in FOF in people with PD [24], and it did not include dual tasking as an independent variable. A recent meta-analysis showed that dual tasking have detrimental effects on gait speed in people with PD [42]. Several studies have shown that dual task training improves dual task performance in people with PD [43–45]; those that seem to benefit the most are those with a slow gait speed (while dual tasking) but with a good cognitive functioning [46]. A highly challenging balance intervention that included dual task training, however, detected any short-term effects neither on gait performance during dual tasking nor on concerns about falling [47]. Still, our findings suggest that dual task training may be of importance when targeting concerns about falling in people with PD.

This study showed that needing help in daily activities predicts concerns about falling, which is in line with prior cross-sectional studies [8, 10, 20]. A recent meta-analysis concluded that functional task training could positively influence activities of daily living (ADL) according to the UPDRS [48], which also applied for highly challenging balance exercises although the effect was small [47]. Our results suggest that one should promote independence in ADL if the primary outcome is concerns about falling. It needs to be noted that dependence in ADL did not significantly predict a change in concerns about falling.

The present study found a trend towards depressive symptoms being a predictive factor of concerns about falling. This is in line with the cross-sectional study by Jonasson et al. [22], which was based on baseline values of the same cohort as ours. Another cross-sectional study identified depressive symptoms as a significant contributing factor to concerns about falling [23]. Studies that instead addressed fall-related self-efficacy have shown contradictory results regarding whether depressive symptoms were independently associated with FOF [20, 21]. The current study showed that depressive symptoms do not predict a change in concerns about falling. To summarize, there are conflicting results whether depressive symptoms contribute to FOF in people with PD. Still, depressive symptoms can have detrimental effects for the person with PD.

Age predicted concerns about falling and both age and female sex predicted a change in concerns about falling. These findings are in contrast to the longitudinal study by Gazibara et al. [24]. Gazibara et al. studied predictive factors of fall-related self-efficacy whereas we studied concerns about falling; this might explain the differences. Although our sample and the sample of Gazibara et al. included the same amount of women (35% vs 34%), our sample was somewhat older (median 69 vs. 60 years of age). This might be another reason for the conflicting results. Our results indicate that rehabilitation efforts that target concerns about falling might be of specific importance for older women with PD, although rehabilitation efforts should be offered at an early time point.

In our study, a history of falls did not predict concerns about falling. This finding agrees with the longitudinal study by Gazibara et al. [24], which addressed fall-related self-efficacy, using dichotomous fall data (although the same study did find an effect when testing the total number of falls, measured at monthly telephone interviews). Moreover, several cross-sectional studies that used multivariable analyses reported nonsignificant associations between fall history and FOF [8, 10, 19, 22].

### 4.1. Methodological Considerations

In order to identify predictive factors, a longitudinal study design is needed. To the best of our knowledge, this is the first study that identified predictive factors of concerns about falling and a change in concerns about falling in people with PD. We included 17 independent variables into an initial linear regression model. Although one could argue that the number of variables in the initial model was somewhat high in relation to the sample size [49], we found the independent variables theoretically important to study. Moreover, the independent variables that were removed in the first steps were clearly nonsignificant, which supports that they should not be part of the final model.

People with cognitive problems were included as long as they were able to give informed consent and take part in the majority of the data collection. Global cognition (i.e., MoCA scores) did not predict concerns about falling, but one should be aware of the fact that there is a debate how cognitive impairments relate to FOF. It might be that cognitive impairments generate insensitivity to FOF, which might increase the risk for future falls [50, 51].

All follow-up studies are at risk of having participants who dropout, this was also the case in the current study which can affect the external validity of the findings.

## 5. Conclusions

This study pinpoints several predictive factors of FOF in people with PD, that are modifiable and which, thereby, could be addressed in rehabilitation: perceived walking difficulties, having problems maintaining balance while dual tasking, and dependence of others in daily activities. Perceived walking difficulties was the strongest predictor for concerns about falling. Problems maintaining balance while dual tasking was also an important predictor and was the strongest predictor for a change in concerns about falling after a 3-year period. This is a novel finding, which needs to be confirmed by future studies.

One should be aware of the fact that an increased age predicts concerns about falling and both age and female sex predicts a change of concerns about falling over time.

## Figures and Tables

**Table 1:** Participants' characteristics at baseline and univariable linear regression analyses with FES-I scores at the 3-year follow-up as the dependent variable, *N* = 151.

Independent variables	Mean	Missing	Univariable regression analyses	
	(SD)	*n*	*B* (95% CI); *β*	*p* value
Age (years)	68 (9.0)	—	0.629 (0.397, 0.860); 0.403	<0.001
Sex (women = 1), *n* (%)	53 (35.1)	—	6.28 (1.64, 11.0); 0.214	0.008
Motor symptoms (UPDRS III)	28.9 (12.4)	—	0.380 (0.207, 0.553); 0.335	<0.001
Fluctuations (yes), *n* (%)	92 (61.7)	2	4.08 (−0.549, 8.70); 0.142	0.084
ADL: needing help (PADLS, yes), *n* (%)	33 (21.9)	—	16.2 (11.4, 21.1); 0.481	<0.001
Mobility device used outdoors (yes), *n* (%)	69 (45.7)	—	12.3 (8.24, 16.5); 0.439	<0.001
Walking difficulties (Walk-12G)	14.8 (10.6)	2	0.900 (0.743, 1.06); 0.683	<0.001
History of falls past 6 months (yes), *n* (%)	64 (42.7)	1	6.72 (2.25, 11.2); 0.237	0.003
Freezing of gait (FOGQsa i.3, yes), *n* (%)	79 (52.3)	—	13.0 (9.00, 17.1); 0.465	<0.001
Dual task: balance problems (yes), *n* (%)	93 (61.6)	—	14.6 (10.5, 18.7); 0.508	<0.001
Orthostatism (NMSQuest i.20, yes), *n* (%)	78 (51.7)	—	7.70 (3.34, 12.1); 0.275	0.001
Living alone (yes), *n* (%)	30 (19.9)	—	5.21 (−0.398, 10.9); 0.149	0.068
Cognitive functioning (MoCA)	25.7 (3.12)	2	−1.33 (−2.02, −0.637); −0.298	<0.001
General self-efficacy (GSE)	29.7 (6.19)	1	−0.811 (−1.15, −0.466); −0.357	<0.001
Pain (yes), *n* (%)	97 (64.2)	—	8.37 (3.84, 12.9); 0.287	<0.001
Depressive symptoms (GDS-15)	2.69 (2.84)	4	2.05 (1.30, 2.80); 0.411	<0.001
Fatigue (NHP-EN, yes), *n* (%)	79 (52.3)	—	10.5 (6.26, 14.7); 0.374	<0.001

FES-I = Falls Efficacy Scale-International (16–64, higher = worse); UPDRS = unified Parkinson's disease rating scale (part III = motor examination, 0–108, higher = worse); ADL = activities of daily living; PADLS = Parkinson's disease ADL scale (those who scored >2 were classified as needing help from others in daily activities); Walk-12G = generic walk-12 (0–42, higher = worse); FOGQsa = self-administered version of the freezing of gait questionnaire, (those who scored >0 were classified as having freezing of gait); NMSQuest = nonmotor symptoms questionnaire; MoCA = Montreal Cognitive Assessment (0–30, higher = better); GSE = general self-efficacy scale (10–40, higher = better); GDS-15 = geriatric depression scale (0–15, higher = worse); NHP-EN = energy subscale of the Nottingham health profile (those who affirmed at least one out of three dichotomous questions were classified as having fatigue). *i* = item number. All dichotomous variables are scored as 0 or 1 (1 = yes).

**Table 2:** FES-I scores (including Cronbach *α* and SEM), *N* = 151.

FES-I item	Baseline mean (SD)	3-year follow-up mean (SD)
1. Cleaning the house	1.84 (1.03)	2.17 (1.14)
2. Getting dressed/undressed	1.60 (0.749)	1.91 (1.01)
3. Preparing simple meals	1.46 (0.728)	1.79 (0.982)
4. Taking a bath or shower	1.63 (0.861)	1.91 (1.05)
5. Going to the shop	1.62 (0.895)	2.00 (1.16)
6. Getting in or out of a chair	1.74 (0.875)	1.95 (0.958)
7. Going up or down stairs	1.76 (0.985)	2.10 (1.09)
8. Walking around outside	1.62 (0.885)	1.88 (1.03)
9. Reaching up or bending down	1.90 (0.998)	2.22 (1.10)
10. Answering the telephone	1.54 (0.755)	1.85 (1.03)
11. Walking on a slippery surface	2.36 (1.05)	2.67 (1.11)
12. Visiting a friend/relative	1.56 (0.829)	1.77 (0.925)
13. Walking in a place with crowds	1.83 (0.929)	2.09 (1.05)
14. Walking on an uneven surface	2.05 (0.968)	2.46 (1.09)
15. Walking up or down a slope	1.96 (1.03)	2.36 (1.13)
16. Going out to a social event	1.69 (0.891)	1.93 (1.02)
FES-I total score	28.1 (11.9)	33.1 (14.0)^1^
Internal consistency (Cronbach *α*)	0.967	0.970
Standard error of measurement (SEM)	2.36	2.25

FES-I = Falls Efficacy Scale-International. Possible item score ranges from 1 to 4, possible total score ranges from 16 to 64, higher = worse. SEM = SD_pooled_ × $\sqrt{1-\text{Cronbach's }\alpha }$. SD_pooled_ = $\sqrt{\left(\left({\text{SD}}_{\text{baseline}}^{2}+{\text{SD}}_{3-\text{year}}^{2}\right)/2\right)}$. ^1^*p* < 0.001, Paired samples *t* test.

**Table 3:** Multivariable linear regression analyses with FES-I (at 3-year follow-up) as the dependent variable: model I (unadjusted for FES-I scores at baseline), *n* = 145.

Independent variables^a^	*B* (95% CI)	*p* value	*β* (standardized *B*)
Walking difficulties (walk-12G)	0.506 (0.284, 0.728)	**<0.001**	0.378
Age (years)	0.355 (0.175, 0.534)	**<0.001**	0.227
Dual task: balance problems (yes = 1)	4.96 (0.967, 8.95)	**0.015**	0.172
ADL: needing help (PADLS, yes = 1)	5.86 (1.37, 10.4)	**0.011**	0.171
Depressive symptoms (GDS-15)	0.595 (−0.025, 1.22)	0.060	0.118
Sex (woman = 1)	3.17 (−0.136, 6.47)	0.060	0.107
	*R* square 59.3%; adjusted *R* square 57.5%		

FES-I = Falls Efficacy Scale-International; walk-12G = generic walk-12 (0–42, higher = worse); ADL = activities of daily living; PADLS = Parkinson's disease ADL scale (those who scored >2 were classified as needing help from others in daily activities); GDS-15 = geriatric depression scale (0–15, higher = worse). ^a^The following 17 independent variables were included in the initial model: age; sex; severity of parkinsonian motor symptoms; motor fluctuations; need help in ADL; use of mobility device outdoors; walking difficulties; a history of falls; freezing of gait; balance problems while dual tasking; orthostatism; living alone; cognitive functioning; general self-efficacy; pain; depressive symptoms; and fatigue. Statistically significant *p* values are bolded.

**Table 4:** Multivariable linear regression analyses with FES-I (at 3-year follow-up) as the dependent variable: model II (adjusted for FES-I scores at baseline), *N* = 151.

Independent variables^a^	*B* (95% CI)	*p* value	*β* (standardized *B*)
Dual task: balance problems (yes = 1)	4.62 (1.19, 8.05)	**0.009**	0.161
Age (years)	0.204 (0.038, 0.371)	**0.017**	0.131
Sex (woman = 1)	3.07 (0.098, 6.05)	**0.043**	0.105
ADL: needing help (PADLS, yes = 1)	3.93 (−0.296, 8.15)	0.068	0.116
	*R* square 63.8%; adjusted *R* square 62.6%		

FES-I = Falls Efficacy Scale-International; ADL = activities of daily living; PADLS = Parkinson's disease ADL scale (those who scored >2 were classified as needing help from others in daily activities). ^a^The following 17 independent variables were included in the initial model: age; sex; severity of parkinsonian motor symptoms; motor fluctuations; need help in ADL; use of mobility device outdoors; walking difficulties; a history of falls; freezing of gait; balance problems while dual tasking; orthostatism; living alone; cognitive functioning; general self-efficacy; pain; depressive symptoms; and fatigue. Statistically significant *p* values are bolded.

## Data Availability

All data are archived according to the Swedish Act concerning the Ethical Review of Research Involving Humans to attain confidentiality. Data can be shared with a qualified researcher upon reasonable request following approval by the responsible ethical committee.

## References

[1] Tinetti M. E., Powell L. (1993). Fear of falling and low self-efficacy: a cause of dependence in elderly persons. *Journal of Gerontology*.

[2] Yardley L., Beyer N., Hauer K., Kempen G., Piot-Ziegler C., Todd C. (2005). Development and initial validation of the falls efficacy scale-international (FES-I). *Age and Ageing*.

[3] Tinetti M. E., Richman D., Powell L. (1990). Falls efficacy as a measure of fear of falling. *Journal of Gerontology*.

[4] Powell L. E., Myers A. M. (1995). The activities-specific balance confidence (ABC) scale. *The Journals of Gerontology Series A: Biological Sciences and Medical Sciences*.

[5] Yardley L., Smith H. (2002). A prospective study of the relationship between feared consequences of falling and avoidance of activity in community-living older people. *The Gerontologist*.

[6] Adkin A. L., Frank J. S., Jog M. S. (2003). Fear of falling and postural control in Parkinson’s disease. *Movement Disorders*.

[7] Camicioli R., Majumdar S. R. (2010). Relationship between mild cognitive impairment and falls in older people with and without Parkinson’s disease: 1-year prospective cohort study. *Gait & Posture*.

[8] Nilsson M. H., Hariz G. M., Iwarsson S., Hagell P. (2012). Walking ability is a major contributor to fear of falling in people with Parkinson’s disease: implications for rehabilitation. *Parkinson’s Disease*.

[9] Jonasson S. B., Nilsson M. H., Lexell J. (2014). Psychometric properties of four fear of falling rating scales in people with Parkinson’s disease. *BMC Geriatrics*.

[10] Lindholm B., Hagell P., Hansson O., Nilsson M. H. (2014). Factors associated with fear of falling in people with Parkinson’s disease. *BMC Neurology*.

[11] Farombi T. H., Owolabi M. O., Ogunniyi A. (2016). Falls and their associated risks in Parkinson’s disease patients in Nigeria. *Journal of Movement Disorders*.

[12] Matinolli M., Korpelainen J. T., Korpelainen R., Sotaniemi K. A., Matinolli V.-M., Myllylä V. V. (2009). Mobility and balance in Parkinson’s disease: a population-based study. *European Journal of Neurology*.

[13] Mak M. K. Y., Pang M. Y. C. (2009). Fear of falling is independently associated with recurrent falls in patients with Parkinson’s disease: a 1-year prospective study. *Journal of Neurology*.

[14] Ellis T., Boudreau J. K., DeAngelis T. R. (2013). Barriers to exercise in people with Parkinson disease. *Physical Therapy*.

[15] Jonasson S. B., Nilsson M. H., Lexell J., Carlsson G. (2018). Experiences of fear of falling in persons with Parkinson’s disease—a qualitative study. *BMC Geriatrics*.

[16] Bryant M. S., Rintala D. H., Hou J.-G., Protas E. J. (2015). Relationship of falls and fear of falling to activity limitations and physical inactivity in Parkinson’s disease. *Journal of Aging and Physical Activity*.

[17] Thordardottir B., Nilsson M. H., Iwarsson S., Haak M. (2014). “You plan, but you never know”—participation among people with different levels of severity of Parkinson’s disease. *Disability and Rehabilitation*.

[18] Brozova H., Stochl J., Roth J., Ruzicka E. (2009). Fear of falling has greater influence than other aspects of gait disorders on quality of life in patients with Parkinson’s disease. *Neuroendocrinology Letters*.

[19] Mak M. K., Pang M. Y., Mok V. (2012). Gait difficulty, postural instability, and muscle weakness are associated with fear of falling in people with Parkinson’s disease. *Parkinson’s Disease*.

[20] Rahman S., Griffin H. J., Quinn N. P., Jahanshahi M. (2011). On the nature of fear of falling in Parkinson’s disease. *Behavioural Neurology*.

[21] Gazibara T., Stankovic I., Tomic A. (2013). Validation and cross-cultural adaptation of the falls efficacy scale in patients with Parkinson’s disease in Serbia. *Geriatrics & Gerontology International*.

[22] Jonasson S. B., Ullén S., Iwarsson S., Lexell J., Nilsson M. H. (2015). Concerns about falling in Parkinson’s disease: associations with disabilities and personal and environmental factors. *Journal of Parkinson’s Disease*.

[23] Franzén E., Conradsson D., Hagstromer M., Nilsson M. H. (2016). Depressive symptoms associated with concerns about falling in Parkinson’s disease. *Brain and Behavior*.

[24] Gazibara T., Tepavcevic D. K., Svetel M. (2019). Change in fear of falling in Parkinson’s disease: a two-year prospective cohort study. *International Psychogeriatrics*.

[25] Nilsson M. H., Iwarsson S. (2013). Home and health in people ageing with Parkinson’s disease: study protocol for a prospective longitudinal cohort survey study. *BMC Neurology*.

[26] Kader M., Ullén S., Iwarsson S., Odin P., Nilsson M. H. (2017). Factors contributing to perceived walking difficulties in people with Parkinson’s disease. *Journal of Parkinson’s Disease*.

[27] Kader M., Jonasson S. B., Iwarsson S., Odin P., Nilsson M. H. (2018). Mobility device use in people with Parkinson’s disease: a 3-year follow-up study. *Acta Neurologica Scandinavica*.

[28] Hoehn M. M., Yahr M. D. (1967). Parkinsonism: onset, progression, and mortality. *Neurology*.

[29] Schwarzer R. J. M., Weinman J., Wright S., Johnston M. (1995). Generalized self-efficacy scale. *Measures in Health Psychology: A User’s Portfolio. Causal and Control Beliefs*.

[30] Fahn S., Elton R. L., Fahn S. (1987). Unified Parkinson’s disease rating scale. *Recent Developments in Parkinson’s Disease*.

[31] Nasreddine Z. S., Phillips N. A., BÃ©dirian V. (2005). The montreal cognitive assessment, MoCA: a brief screening tool for mild cognitive impairment. *Journal of the American Geriatrics Society*.

[32] Bladh S., Nilsson M. H., Hariz G.-M., Westergren A., Hobart J., Hagell P. (2012). Psychometric performance of a generic walking scale (Walk-12G) in multiple sclerosis and Parkinson’s disease. *Journal of Neurology*.

[33] Hunt S. M., McKenna S. P., McEwen J., Backett E. M., Williams J., Papp E. (1980). A quantitative approach to perceived health status: a validation study. *Journal of Epidemiology & Community Health*.

[34] Hagell P., Höglund A., Reimer J. (2006). Measuring fatigue in Parkinson’s disease: a psychometric study of two brief generic fatigue questionnaires. *Journal of Pain and Symptom Management*.

[35] Hobson J. P., Edwards N. I., Meara R. J. (2001). The Parkinson’s disease activities of daily living scale: a new simple and brief subjective measure of disability in Parkinson’s disease. *Clinical Rehabilitation*.

[36] Nilsson M. H., Hariz G. M., Wictorin K., Miller M., Forsgren L., Hagell P. (2010). Development and testing of a self administered version of the freezing of gait questionnaire. *BMC Neurology*.

[37] Chaudhuri K. R., Martinez-Martin P., Schapira A. H. V. (2006). International multicenter pilot study of the first comprehensive self-completed nonmotor symptoms questionnaire for Parkinson’s disease: the NMSQuest study. *Movement Disorders*.

[38] Sheikh J. A., Yesavage J. A., Sheikh J. (1986). Geriatric depression scale (GDS): recent evidence and development of a shorter version. *Clinical Gerontology: A Guide to Assessment and Intervention*.

[39] Mehrholz J., Kugler J., Storch A., Pohl M., Hirsch K., Elsner B. (2015). Treadmill training for patients with Parkinson’s disease. *Cochrane Database of Systematic Reviews*.

[40] Keus SHJ M. M., Graziano M. (2014). *European Physiotherapy Guideline for Parkinson’s Disease*.

[41] Cakit B. D., Saracoglu M., Genc H., Erdem H. R., Inan L. (2007). The effects of incremental speed-dependent treadmill training on postural instability and fear of falling in Parkinson’s disease. *Clinical Rehabilitation*.

[42] Raffegeau T. E., Krehbiel L. M., Kang N. (2019). A meta-analysis: Parkinson’s disease and dual-task walking. *Parkinsonism & Related Disorders*.

[43] Brauer S. G., Morris M. E. (2010). Can people with Parkinson’s disease improve dual tasking when walking?. *Gait & Posture*.

[44] Fritz N. E., Cheek F. M., Nichols-Larsen D. S. (2015). Motor-cognitive dual-task training in persons with neurologic disorders: a systematic review. *Journal of Neurologic Physical Therapy*.

[45] Strouwen C., Molenaar E. A. L. M., Münks L. (2017). Training dual tasks together or apart in Parkinson’s disease: results from the DUALITY trial. *Movement Disorders*.

[46] Strouwen C., Molenaar E. A. L. M., Münks L. (2019). Determinants of dual-task training effect size in Parkinson disease: who will benefit most?. *Journal of Neurologic Physical Therapy*.

[47] Conradsson D., Löfgren N., Nero H. (2015). The effects of highly challenging balance training in elderly with Parkinson’s disease: a randomized controlled trial. *Neurorehabilitation and Neural Repair*.

[48] Perry S. I. B., Nelissen P. M., Siemonsma P., Lucas C. (2019). The effect of functional-task training on activities of daily living for people with Parkinson’s disease, a systematic review with meta-analysis. *Complementary Therapies in Medicine*.

[49] Harrell F. E. (2015). *Regression Modeling Strategie: With Applications to Linear Models, Logistic and Ordinal Regression, and Survival Analysis*.

[50] Uemura K., Shimada H., Makizako H. (2012). A lower prevalence of self-reported fear of falling is associated with memory decline among older adults. *Gerontology*.

[51] Shirooka H., Nishiguchi S., Fukutani N. (2017). Cognitive impairment is associated with the absence of fear of falling in community-dwelling frail older adults. *Geriatrics & Gerontology International*.

